# A Novel Mutation in Chronic Granulomatous Disease: Treating the Family, Not Just the Patient

**DOI:** 10.3389/fped.2019.00107

**Published:** 2019-03-28

**Authors:** Kristen Lutzkanin, Daniel J. McKeone, Robert Greiner, Doerthe Adriana Andreae

**Affiliations:** ^1^Division of Pediatric Allergy and Immunology, Department of Pediatrics, Penn State Milton S. Hershey Medical Center, Hershey, PA, United States; ^2^Division of Pediatric Hematology and Oncology, Department of Pediatrics, Penn State Milton S. Hershey Medical Center, Hershey, PA, United States

**Keywords:** chronic granulomatous disease, primary innate immunodeficiency, NAPDH oxidase, dihydrorhodamine test, stem cell transplantation

## Abstract

Chronic Granulomatous Disease (CGD) is caused by genetic defects in the phagocyte NADPH oxidase leading to potentially severe infections with catalase positive micro-organisms. With the innate immune system being affected this disease usually presents before the age of 5 years with infections involving the skin, lung, liver or lymphnodes. Infections with specific catalase positive organisms, especially *Burkholderia cepacia, Serratia, Nocardia* and *Chromobacterium violaceum* prompt a workup for CGD in affected patients. In addition, a family history of CGD also warrants testing. The pattern of inheritance of CGD varies across geographic regions of the world and societies, with X-linked inheritance being most prevalent in the United States and Europe. Affected patients require life-long therapy with prophylactic antibiotics, antifungals, and possibly interferon-gamma. Hematopoietic Stem Cell Transplantation is the only curative therapy known to date. Identification, diagnosis and management of patients with CGD usually involves a multi-specialty team including Pediatrics, Immunology, Infectious Diseases, Hematology/Oncology and often also Pulmonology and GI/Hepatology. Frequent follow up is paramount for good outcomes; infections have to be recognized and treated promptly and often preemptively. This is challenging for most patients and their families but presents a significant barrier for patients with limited access to care, limited resources or other challenging social situations. This case report describes the difficulties of managing a family with a novel mutation and multiple affected family members in different custody arrangements. It highlights the importance of close contact and communication with the family in deciding on management and treatment options. Educating the family and patient is critical to avoid complications of the disease and allow shared decision making that ultimately leads to better outcomes.

## Introduction

Chronic granulomatous disease (CGD) is a primary innate immunodeficiency caused by genetic defects in the phagocyte NADPH oxidase (1). CGD is characterized by infections with catalase-positive micro-organisms, which includes most bacterial and all fungal organisms (2). It is a rare disorder, with an incidence of 1 in 200,000 to 1 in 250,000 live births in the United States (3). In the United States and Europe, CGD is typically inherited in an X-linked pattern. Approximately 30% of cases are inherited in an autosomal recessive pattern, with a higher incidence in certain geographical regions (4).

There are five subunits which comprise the NADPH oxidase enzyme, with two membrane-bound components (gp91^*phox*^ and p22^phox^) and three cytoplasmic components (p47^*phox*^, p67^*phox*^, and p40^*phox*^) which are all necessary for the generation of the superoxide. The membrane-bound components, gp91^*phox*^ and p22^phox^ together form a single subunit known as cytochrome b_558_ which is embedded in the walls of the secondary granules_._ When functioning normally, the membrane-bound and cytoplasmic subunits come together on the phagolysosome surface to form the NADPH oxidase initiated by the phagocytosis of microbes. The functioning enzyme then catalyzes the transfer of an electron from NADPH in the cytoplasm to molecular oxygen within the phagolysosome. The process is responsible for creating superoxide radicals which then combine with water within the phagolysosome to generate reactive oxygen species such as superoxide and its metabolites which is converted to hydrogen peroxide. In the final step hypochlorous acid and chlorine are produced which exhibit toxic effects on phagocytosed organisms (5).

CGD is caused by defects in any of the five subunits. Mutations in any structural subunit of the NADPH oxidase enzyme disrupts the catalytic pathway responsible for generating reactive oxygen species, therefore leading to defective microbial killing. Defects in gp91^*phox*^ are most common, accounting for ~70% of cases of CGD. This protein is encoded by the *CYBB* gene located on the X chromosome at Xp21.1. There have been more than 500 mutations described in the *CYBB* gene which are responsible for CGD. Defects in p47^*phox*^ account for ~20–25% of cases. This protein is encoded by the *NCF1* gene is located on chromosome 7q11.23. Defects in p67^*phox*^ (on chromosome 1q25) and p22^phox^ (on chromosome 16q24) account for the remaining ~5% of CGD cases (4). These proteins are encoded by the *NCF2* and *CYBA* genes, respectively. There is one documented case of CGD resulting from a p40^phox^ deficiency encoded by *NCF4* (6).

Other defects have also been reported to cause CGD which are not mutations in the structural subunits of the NADPH oxidase. Recently a homozygous loss of function mutation in *CYBC1* has also been reported as a possible cause of CGD, the gene product of which is thought to be a chaperone protein which is important in the formation of the NADPH oxidase (7).

CGD is typically diagnosed in childhood, with most patients presenting prior to the age of five (8, 9). Due to the defective oxidative burst, patients with CGD are susceptible to infections with catalase positive organisms such as *Aspergillus, S. aureus, Burkholderia cepacia, Serratia, Nocardia* (2)*. Salmonella* and mycobacteria may also be sources of infection, particularly outside of the United States (10). Classic sites of infection include the skin (abscesses), lungs (pneumonia), lymph nodes (adenitis), and liver (abscesses). Staphylococcal infections typically involve the liver and lymph nodes. Infection with *Burkholderia spp*. is more likely to present as pneumonia or sepsis and by itself is highly suspicious for CGD or cystic fibrosis. Infants presenting with *Serratia spp*. infections may have osteomyelitis or soft tissue infections, whereas older children and adults may present with skin infections. *Nocardia spp*. infection, which is rare outside of CGD, typically presents as pneumonia, but may also include osteomyelitis or brain abscesses (2). Infection with *Chromobacterium violaceum* is rare, but suspicion must be high for CGD in a patient with this infection which is typically obtained from swimming in brackish water (11).

Other than infections, patients with CGD may experience poor growth, poor wound healing, and diarrhea. They are at risk for other inflammatory conditions including granuloma formation, inflammatory bowel disease, and genitourinary complications (1).

Infection with any of the above mentioned organisms or history of recurrent classic types of infection without a named organism should prompt a workup for CGD. The diagnosis of CGD can be confirmed by performing the dihydrorhodamine test, a flow cytometry assay (12). Historically, the nitroblue tetrazolium test (NBT) was an *in vitro* assay performed in which normal activated neutrophils would produce superoxide turning yellow NBT to blue, a rapid, but only qualitative assay for CGD (13). Today, dihydrorhodamine (DHR) is the test of choice for diagnosing CGD. In this test, NADPH oxidase activity is quantified by measuring DHR oxidization to a green fluorescent compound (12, 14). DHR is a more sensitive test than NBT and can help distinguish between X-linked defects, autosomal defects or CGD carriers. An abnormal DHR should be followed up by genetic testing.

Once the diagnosis of CGD is confirmed, prophylactic treatment targeted at the classic organisms implicated in CGD must be initiated. Prophylactic trimethoprim-sulfamethoxazole (5 mg/kg/day up to 320 mg) and itraconazole (100 mg/day <13 year/50 kg or 200 mg/day >13 year/50 kg) are the medications of choice. Prophylactic antimicrobials have changed the disease course of CGD. Prior to oral antifungal prophylaxis, the mortality rate for CGD was reported as 5% per year for X-CGD. Survival rates are currently estimated at 90% at 10 years (2, 8, 9, 12).

Interferon gamma is part of the prophylactic regimen in some centers in the United States. It is less commonly used in other parts of the world. Studies investigation the effect and mechanism are underway. At this time the mechanism and target group within CGD is not well-described, but it appears that interferon gamma reduces infections, particularly pneumonia, in patients with CGD (15).

Patients with CGD should be evaluated for possible hematopoietic stem cell transplant (HSCT). With the advent of prophylactic antibiotics, interferon gamma treatment and HSCT, the survival rate for CGD ~90% at 10 years (16).

Allogenic hematopoietic stem cell transplantation (HSCT) is the only known curative treatment option for CGD. There is increasing evidence that HSCT is preferable to conventional infectious prophylaxis in severe CGD when an appropriate donor source is available (16). The best reported outcomes are with fully matched siblings that are unaffected by CGD. Other acceptable stem cell sources are from HLA-matched unrelated donors (MUD) as well as umbilical cord blood. Excellent outcomes using myeloablative conditioning (MAC) have been described. However, these regimens are associated with significant long-term effects and are less attractive for patients with significant comorbidities (17).

Reduced-intensity conditioning (RIC) regimens have been increasingly utilized for the treatment of non-malignant diseases such as CGD (18). RIC carries a more favorable transplant-related toxicity profile, including decreased risk of acute graft-vs.-host disease (GvHD) as well as late effects (19). Survival outcomes from RIC are similar to MAC although the risk of graft failure is higher with RIC, especially when using umbilical cord stem cells (20). Collection of CD34+ stem cells from marrow or peripheral blood donors remains an option for treatment of graft failure whereas this is not currently possible with umbilical cord blood.

Gene therapy is a potential option for patients without an appropriate HLA-matched donor. Early trials were complicated by lack of sustained therapeutic response and insertional mutagenesis resulting in myelodysplastic syndrome. However, subsequent trials with new approaches have shown promising results (21).

## Case Report

A 19 month old male presented to the office for evaluation of recurrent ear infections. His mother reported three to four infections which required antibiotics. He had required extended courses of antibiotics, but not IV antibiotics. There was no history of pneumonia, skin abscesses, suppurative adenitis, osteomyelitis, sepsis (bacterial or fungal), or cellulitis/impetigo. He was otherwise healthy with a history of mild atopic dermatitis.

On exam, his vitals were within normal limits for age. His physical exam was unremarkable except for dry, erythematous patches behind the knees, bilateral antecubital fossa, and axilla.

At the time of initial presentation the patient was living with his biological mother, and two half-brothers. Two older half-brothers were removed from his mother's care prior. He did not attend day care. Mother denied consanguinity with his biological father; in addition, all of her five children had different fathers. There were no pets in the home.

**Figure 1:**
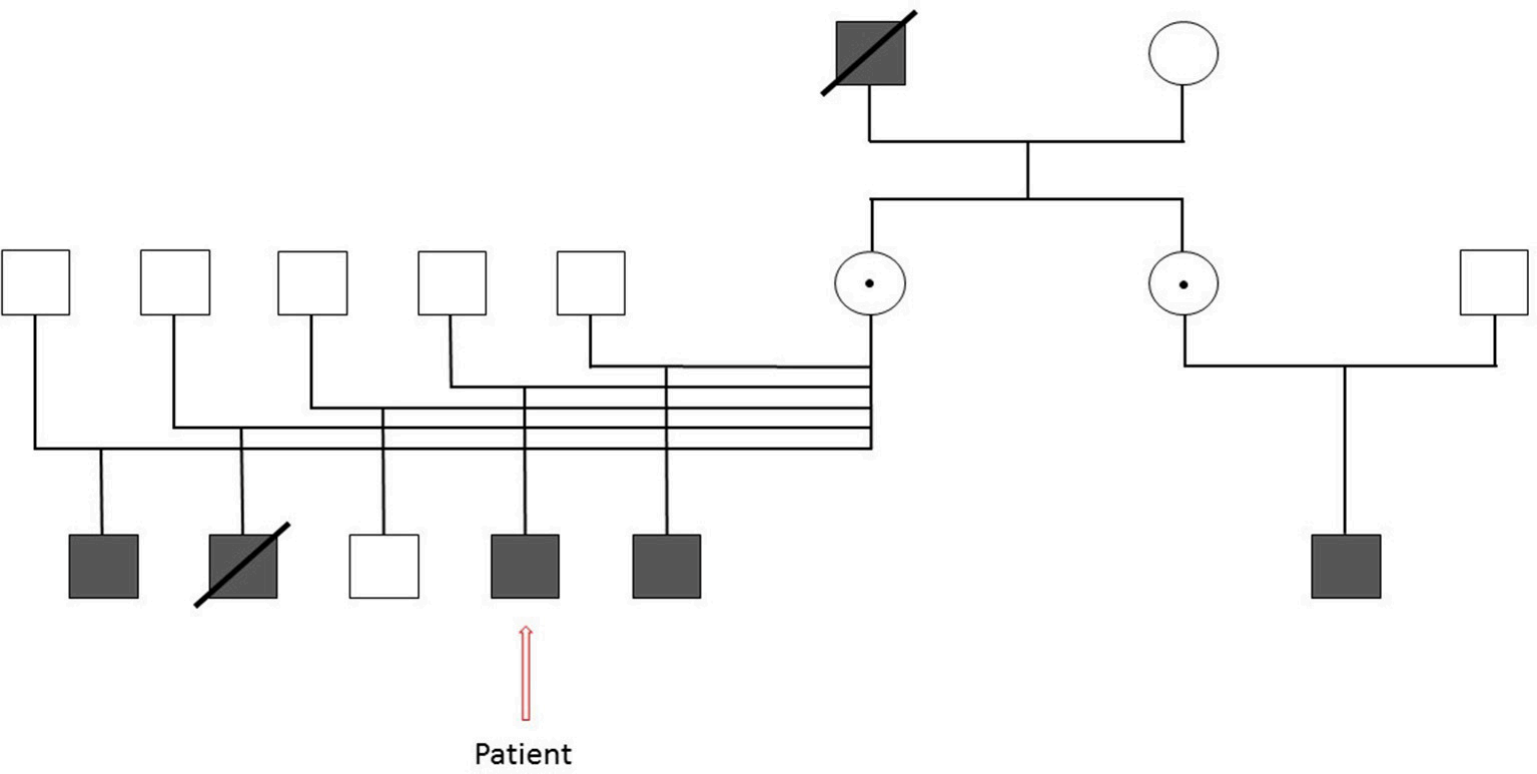
Extended family pedigree of our patient with Chronic Granulomatous Disease illustrating the extensive family history of the disease. Square- Male, Darkened square- Affected male, Circle- Female, Dotted Circle- Carrier, Strikethrough- Deceased.

Family history was significant for serious infections in other family members. The patient's maternal grandfather died at an early age (around age 30 years) due to pneumonia. A half-brother in foster care with a history of severe infections was diagnosed with CGD based on DHR. Genetic testing revealed a novel c141+4 A>G mutation of the CYBB gene that is not a common benign variant in individuals with European or African American ancestry. Two of three *in-silico* splice prediction models (NetGene2, Softberry) predicted damage to the splice donor site for intron 2. A third model (BDGP) predicted no effect on splicing. In the absence of RNA/functional studies, the actual effect of the sequence change is unknown so the mutation was classified as a variant of uncertain significance. A second half-brother in foster care with a different family had been admitted at this academic institution with Burkholderia sepsis and died. While this same child had infection with salmonella at 2 years of age, the diagnosis of CGD was not known at the time of admission. Due to the severity and quick onset of his illness, CGD testing was not obtained. CGD testing could not be completed post-mortem. The diagnosis of CGD was suspected because of his infection with *Burkholderia* spp. The mother was located and testing confirmed that she was an X-linked carrier of CGD. Based on this result our patient and his two half-brothers were seen for evaluation and testing in our clinic. Subsequently, his mother learned about the CGD diagnosis of her sister's son (Figure 1).

**Figure 2:**
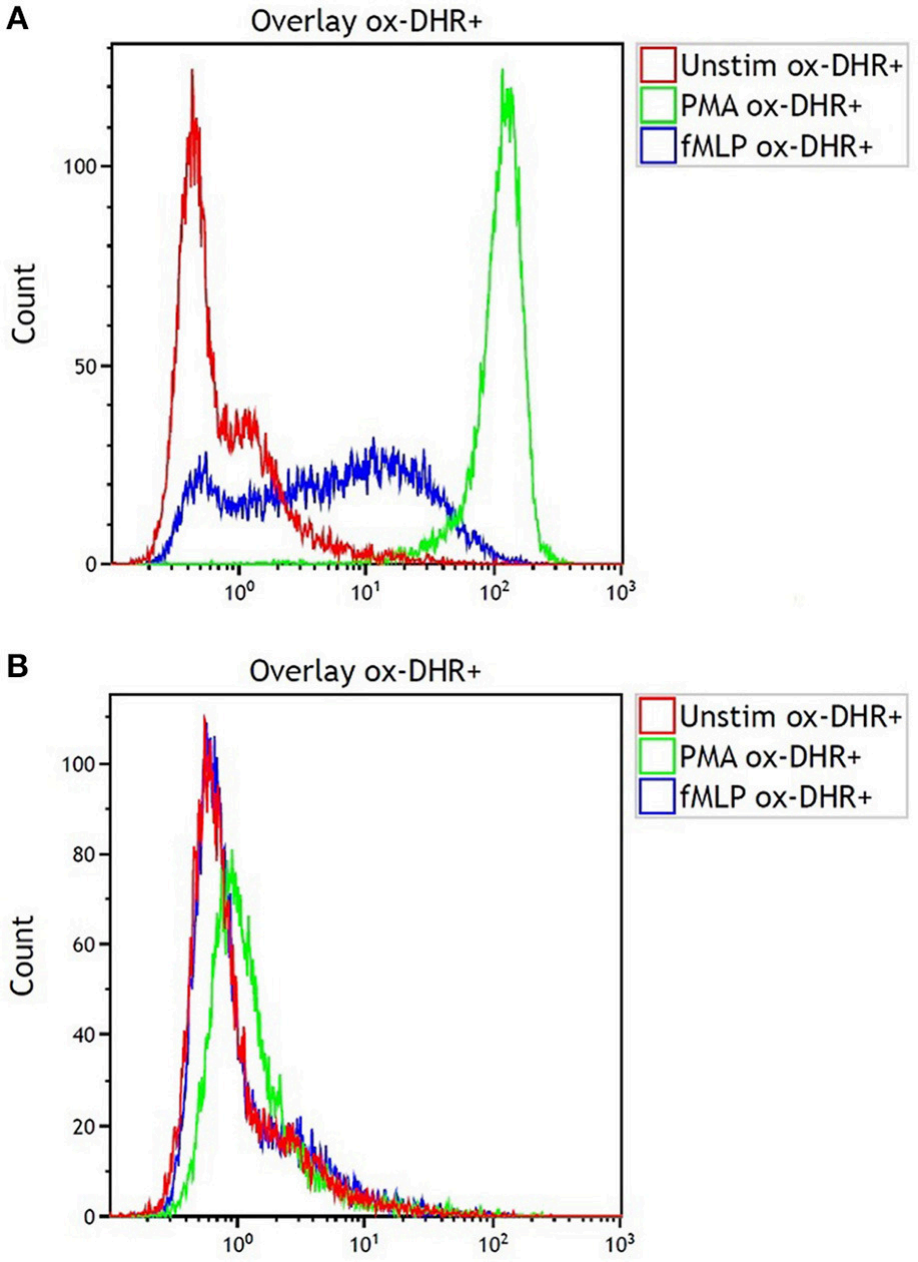
Dihydrorhodamine test results for **(A)** the shipping control and **(B)** our patient with CGD showing fluorescence after incubation of serum sample with dihydrorhodamine and phorbol myristate acetate (PMA). Note the lack of fluorescence shift in B indicating lack of oxidative phosphorylation.

The DHR was grossly abnormal (Figure 2A, control; Figure 2B, patient) consistent with the diagnosis of CGD. One of the two half-brothers also tested positive for CGD, the other tested negative. The patient was started on trimethoprim-sulfamethoxazole and itraconazole prophylaxis and also referred to Hematology/Oncology for HSCT evaluation.

After careful consideration of the risks and benefits, Hematology/Oncology recommended proceeding with HSCT as the only chance of curing the patient's underlying CGD. As previously discussed, RIC regimens are favored over MAC regimens due to the lower risk of transplant-related toxicities and mortality. The use of donor bone marrow or peripheral blood stem cells (PBSCs) as the stem cell source with a RIC regimen is favored over umbilical cord stem cells due to the high risk of graft failure with cord blood transplantation after RIC. Bone marrow sources are preferred over PBSCs due to the lower incidence of chronic graft-vs.-host disease.

As the patient did not have any known full siblings, the next best stem cell source is bone marrow from a highly HLA-matched unrelated donor. The search for a healthy, full-HLA match is ongoing with plans to proceed with HSCT when an appropriate donor is identified.

## Discussion

In CGD, the predominant defect is in the CYBB gene which encodes gp91-phox. There are over 500 mutations reported in the CYBB gene which have accounted for cases of CGD (4). Knowledge of genetic mutations can help in the diagnosis of carriers, assist in genetic counseling and prenatal screening. It is notable that in the case of this family, there is a novel c141+4 A>G mutation which to our knowledge has not been reported. In general the production of different proteins by alternative splicing is very common in humans. The use of next generation sequencing allows for high throughput analysis and identification of gene variants. The major challenge of using *in silico* tools to predict splicing defects lies in the interpretation of the result output. As no standard measure exists regarding the splicing signal, results are often reported as prediction scores which vary depending on method and platform. This also explains the different reported results for our patient. A mutation detected by *in silico* splicing cannot *per se* be classified as pathogenic or benign. In our case, however, the suspicion that this detected mutation in the CYBB gene leads to sufficient decrease of neutrophil oxidative burst is high, as the patient's half-brother who likely carries that same mutation developed a severe lethal infection with Burkholderia (22). Adding this mutation to the existing database can advance scientific knowledge with regard to CGD and help families make knowledgeable decisions regarding family planning. Furthermore, while gene therapy has not been robustly successful in CGD, knowledge of specific genetic mutations may be helpful to advance that field (23).

This case illustrates the importance of a detailed current and past medical history including a family history when evaluating for possible immunodeficiencies. The history can point the clinician toward the correct diagnosis, whether the immunodeficiency is of the innate immune system, humoral immune system or adaptive immune system. The patient age and gender, type of organisms and loci of infection, other associated diseases (including autoimmunity), dysmorphic features, or other syndromic features and family history may all point the clinician to suspect involvement of a particular arm of the immune system or even a specific disease. In this case, his male gender and family history were suggestive of an X-linked autosomal recessive disease, especially based on the fact that each of his affected brothers had a different father. Even without confirmation of CGD in his older half-brother, the organisms and loci of infection were suggestive of CGD. Obtaining these key aspects of the history and narrowing down the differential diagnosis can lead to directed laboratory testing rather than a costly general screen of the immune system. In this case, directed laboratory testing with a DHR was obtained which confirmed the suspicion for CGD.

This case also illustrates the importance of obtaining an accurate social history. It is important to note that in patients with chronic medical conditions, the social history can help you understand the support system the patient/family has in place to cope with the diagnosis. Is the home a safe place to be, or is the patient exposed to possible pathogens in the house? Can the family care for the patient on their own? Can medications and equipment be afforded? Are there certain exposures which place the patient at risk for possible complications? These are all questions that should be answered by a thorough social history. Furthermore, social histories may evolve over time, so it is essential to reassess changes with the patient and family.

Chronic medical conditions can be a great stressor on families. Our patient was living with his mother and two-half siblings. Shortly after being diagnosed with CGD, care of the patient was relinquished to the patient's father without informing the father of the diagnosis and the need for prophylactic medications. It was learned that father was a landscaper, and in his home, they had a pet iguana. These exposures put our patient with CGD at risk for infections. The father was instructed to shower and change clothes prior to interacting with his son after work, and clear instructions were provided to avoid handling the iguana. While all patients with CGD should receive counseling on avoid exposure to mulch or potting soil, this family received specific, directed education based on his environmental exposures.

Another important factor is coordination of care with a primary care provider (PCP). It is important for patients with CGD to have a physician they can speak with/visit for evaluation of fever or other infectious symptoms. This patient did not have an established PCP since transferring from his mother to his father's care. It was essential that our medical team worked to set him up with a provider who could serve as the front line for assessment of possible infections.

This case highlights the importance of a multispecialty, team approach to caring for patients with CGD and consideration of the social situation and environment of the patient and family to achieve the best possible outcome (Table 1).

**Table 1:** Medical and social considerations in patients with CGD.

**Medical Home**
Patient should have access to:
-A single provider/office to oversee and manage the entirety of care (i.e., PCP or social worker)
-PCP familiar with CGD
-Regularly scheduled well-child checks
-Routine immunizations
-Immunologist
-Hematologist/oncologist
-Other subspecialties (ID, GI, Pulmonary)
-Emergency Medical Care at a center familiar with CGD
-Social Worker/Care coordinator to oversee patient's care
-Medicaid for children who do not have primary insurance, or as a secondary insurance
-Local pharmacy to fill medications with adequate supply of refills
**Home/School**
Patient should avoid:
-Handling garden mulch or potting soil (exposure to Aspergillus)
-It is safe to play outside once mulch is firmly on the ground
-Turning manure/compost piles
-Raking leaves
-Barns/hayrides
-Cleaning cellars/garages, demolition
-Swimming in brackish water (exposure *Chromobacterium violaceum)*
-Smoking marijuana (possible exposure to Aspergillus)
-Reptiles (exposure to salmonella)

## Data Availability

The datasets generated for this study are available on request to the corresponding author.

## Ethics Statement

Informed consent was obtained from the patient's father (his legal guardian) to publish the case report.

## Author Contributions

All authors were part of the clinical team caring for the reported patient and involved in clinical decision making. KL wrote the first draft of the manuscript. DM, RG, and DA wrote sections of the manuscript. All authors contributed to manuscript revision, read and approved the submitted version.

### Conflict of Interest Statement

The authors declare that the research was conducted in the absence of any commercial or financial relationships that could be construed as a potential conflict of interest.

## References

[1] Leiding JW Holland SM Chronic Granulomatous Disease. GeneReviews® (1993). 22876374

[2] Marciano BE Spalding C Fitzgerald A Mann D Brown T Osgood S. Common severe infections in chronic granulomatous disease. Clin Infect Dis. (2015) 60:1176–83. 10.1093/cid/ciu1154 25537876 PMC4400412

[3] Winkelstein JA Marino MC Johnston RB Jr Boyle J Curnutte J. Chronic granulomatous disease; report on a national registry of 368 patients. Medicine. (2000) 79:155–69. 10.1016/S0161-5890(98)90515-6 10844935

[4] Roos D Kuhns DB Maddalena A Roesler J Lopez JA Ariga T. Hematologically important mutations: X-linked chronic granulomatous disease (third update). Blood Cells Mol Dis. (2010) 45:246–65. 10.1016/j.bcmd.2010.07.012 20729109 PMC4360070

[5] Segal BH Leto TL Gallin JI Malech HL Holland SM. Genetic, biochemical, and clinical features of chronic granulomatous disease. Medicine. (2000) 79:170–200. 10.1097/00005792-200005000-00004 10844936

[6] Matute JD Arias AA Wright NAM Wrobel I Waterhouse CCM Li XJ. A new genetic subgroup of chronic granulomatous disease with autosomal recessive mutations in p40 phox and selective defects in neutrophil NADPH oxidase activity. Blood. (2009) 114:3309–15. 10.1182/blood-2009-07-231498 19692703 PMC2759653

[7] Arnadottir GA Norddahl GL Gudmundsdottir S Agustsdottir AB Sigurdsson S Jensson BO. A homozygous loss-of-function mutation leading to CYBC1 deficiency causes chronic granulomatous disease. Nat Commun. (2018) 9:4447. 10.1038/s41467-018-06964-x 30361506 PMC6202333

[8] Jones LBKR McGrogan P Flood TJ Gennery AR Morton L Thrasher A. Special article: chronic granulomatous disease in the United Kingdom and Ireland: a comprehensive national patient-based registry. Clin Exp Immunol. (2008) 152:211–8. 10.1111/j.1365-2249.2008.03644.x 18410635 PMC2384093

[9] Martire B Rondelli R Soresina A Pignata C Broccoletti T Finocchi A. Clinical features, long-term follow-up and outcome of a large cohort of patients with Chronic Granulomatous Disease: An Italian multicenter study. Clin Immunol. (2008) 126:155–64. 10.1016/j.clim.2007.09.008 18037347

[10] van JM van Koppen E Ahlin A Belohradsky BH Bernatowska E Corbeel L Chronic granulomatous disease: the European experience. PLoS One. (2009) 4:e5234 10.1371/journal.pone.0005234 19381301 PMC2668749

[11] Sirinavin S Techasaensiri C Benjaponpitak S Pornkul R Vorachit M. Invasive Chromobacterium violaceum infection in children: case report and review. Pediatr Infect Dis J. (2005) 24:559–61. 10.1097/01.inf.0000164761.81491.3f 15933571

[12] Kuhns DB Alvord WG Heller T Feld JJ Pike KM Marciano BE. Residual NADPH Oxidase and Survival in Chronic Granulomatous Disease. N Engl J Med. (2010) 363:2600–10. 10.1056/NEJMoa1007097 21190454 PMC3069846

[13] Baehner RL Nathan DG. Leukocyte oxidase: defective activity in chronic granulomatous disease. Sci Am Assoc Adv Sci. (1967) 155:835–6. 10.1126/science.155.3764.835 6018195

[14] Jirapongsananuruk O Malech HL Kuhns DB Niemela JE Brown MR Anderson-Cohen M. Diagnostic paradigm for evaluation of male patients with chronic granulomatous disease, based on the dihydrorhodamine 123 assay. J Allergy Clin Immunol. (2003) 111:374–9. 10.1067/mai.2003.58 12589359

[15] Loffredo L Perri L Zicari A Del Ben M Angelico F Violi F. Chronic granulomatous disease as an SOS call for multicenter cooperative effort to prevent infections: A meta-analysis of the treatments. Ann Allergy Asthma Immunol. (2016) 117:285–9. 10.1016/j.anai.2016.07.008 27613462

[16] Åhlin A Fasth A. Chronic granulomatous disease – conventional treatment vs. hematopoietic stem cell transplantation. Curr Opin Hematol. (2015) 22:41–5. 10.1097/moh.0000000000000097 25394312

[17] Tewari P Martin PL Mendizabal A Parikh SH Page KM Driscoll TA. Myeloablative transplantation using either cord blood or bone marrow leads to immune recovery, high long-term donor chimerism and excellent survival in chronic granulomatous disease. Biol Blood Marrow Transplant. (2012) 18:1368–77. 10.1016/j.bbmt.2012.02.002 22326631 PMC3540103

[18] Güngör T Teira P Slatter M Stussi G Stepensky P Moshous D. Reduced-intensity conditioning and HLA-matched haemopoietic stem-cell transplantation in patients with chronic granulomatous disease: a prospective multicentre study. Lancet. (2014) 383:436–48. 10.1016/S0140-6736(13)62069-3 24161820

[19] Khandelwal P Bleesing JJ Davies SM Marsh RA. A single-center experience comparing alemtuzumab, fludarabine, and melphalan reduced-intensity conditioning with myeloablative busulfan, cyclophosphamide, and antithymocyte globulin for chronic granulomatous disease. Biol Blood Marrow Transplant. (2016) 22:2011–8. 10.1016/j.bbmt.2016.08.0131083-8791 27543157

[20] Prasad VK. Stem-cell transplantation for chronic granulomatous disease. Lancet. (2014) 383:390–2. 10.1016/S0140-6736(13)62144-3 24161823

[21] Arnold DE Heimall JR. A Review of Chronic Granulomatous Disease. Adv Ther. (2017) 34:2543–57. 10.1007/s12325-017-0636-2 29168144 PMC5709447

[22] Jian X Boerwinkle E Liu X. In silico prediction of splice-altering single nucleotide variants in the human genome. Nucl Acids Res. (2014) 42:13534–44. 10.1093/nar/gku1206 25416802 PMC4267638

[23] Roos D de Boer M. Molecular diagnosis of chronic granulomatous disease. Clin Exp Immunol. (2014) 175:139–49. 10.1111/cei.12202 24016250 PMC3892405

